# Development of TiAl–Si Alloys—A Review

**DOI:** 10.3390/ma14041030

**Published:** 2021-02-22

**Authors:** Anna Knaislová, Pavel Novák, Marcello Cabibbo, Lucyna Jaworska, Dalibor Vojtěch

**Affiliations:** 1 Department of Metals and Corrosion Engineering, University of Chemistry and Technology, Prague, Technická 5, 166 28 Prague, Czech Republic; panovak@vscht.cz (P.N.); vojtechd@vscht.cz (D.V.); 2 DIISM/Università Politecnica delle Marche, Via Brecce Bianche 12, 60131 Ancona, Italy; m.cabibbo@staff.univpm.it; 3 Department of Materials Science and Non-Ferrous Metals Engineering, AGH University of Science and Technology, aleja Adama Mickiewicza 30, 30-059 Krakow, Poland; ljaw@agh.edu.pl

**Keywords:** titanium aluminide, titanium silicide, alloy, synthesis, properties

## Abstract

This paper describes the effect of silicon on the manufacturing process, structure, phase composition, and selected properties of titanium aluminide alloys. The experimental generation of TiAl–Si alloys is composed of titanium aluminide (TiAl, Ti_3_Al or TiAl_3_) matrix reinforced by hard and heat-resistant titanium silicides (especially Ti_5_Si_3_). The alloys are characterized by wear resistance comparable with tool steels, high hardness, and very good resistance to oxidation at high temperatures (up to 1000 °C), but also low room-temperature ductility, as is typical also for other intermetallic materials. These alloys had been successfully prepared by the means of powder metallurgical routes and melting metallurgy methods.

## 1. Introduction

There is currently extensive research on intermetallics for high temperature applications, especially for the automotive, aviation, and cosmic applications [1,2,3]. Titanium aluminides are of the greatest importance for the automotive industry, when turbochargers made of TiAl intermetallic alloys have already started to be used in passenger cars [4]. The use of these alloys in aircraft engines (for low-pressure turbine blades) is very interesting [4,5]. Today’s titanium alloys and titanium aluminides account for a third of the weight of aircraft engines and are the second most widely used material for engine components after nickel superalloys [6].

Cold extreme tool steels or high speed steels are most commonly used today for extreme adhesive and abrasive wear [7,8,9]. High temperature applications use nickel superalloys in aviation-related applications, such as engine turbines. Another widely used material is heat-resistant steels, the great advantage of which is the relatively simple production [10]. However, the problem is their high density [11]. In aviation, there is an effort to replace nickel [12] and heat-resistant steels with lighter materials to decrease the weight of the aircraft structure and to improve engine performance [10,11]. The strength-to-weight ratio could be significantly improved by significantly reducing weight by the means of a substitution of nickel-based superalloys with intermetallic alloys, such as titanium aluminides [6].

Titanium alloys with other light elements (aluminum, silicon) are therefore very promising materials for applications at higher temperatures [13,14], especially for use in structural applications operating under static load and at the same time high temperatures [15]. Their biggest advantage is the low density, which ranges around 4 g·cm^−3^ [16,17].

## 2. History of Using TiAl Alloys

In the second half of the last century, TiAl alloys began to be used as technical materials. Rolls-Royce, P&W, and GE tested automotive engines made from TiAl intermetallic alloy. Advantageous properties of TiAl motors include better specific tensile strength compared to other titanium alloys and higher strength at temperatures up to 800 °C compared to steels or nickel superalloys. However, a major disadvantage is the low room-temperature strain, usually up to 1% (1% is generally an acceptable minimum ductility value, cast samples seldom reach this value). Another problem is the difficult production of the component [18].

In the 1970s, extensive research was carried out on Ti_3_Al-based systems for the production of aircraft engines, but this production failed due to the high difficulty and the limit temperature of use of the component (600 °C) [5]. In the 1980s, research was shifted to γ–TiAl alloys, which had better creep and oxidation resistance, and the maximum application temperature was increased to 750 °C. In addition to aviation, alloys have potential use in the automotive and nuclear industries [5]. The US Air Force then stated that TiAl intermetallics provide unique weight savings as a replacement for nickel superalloys in the hot parts of low-pressure turbine jet engines [19]. However, problems with the use of TiAl alloys in aviation include large deviation of mechanical properties, difficult production and high costs [5]. On the other hand, the use of an aircraft engine made of TiAl alloy reduces the weight from 20% to 50% compared to conventional materials, but production costs increase by 10% to 100% [5].

In this millennium, research has focused on TiAl alloys with 44–48 at. % aluminum, but for some applications, a maximum of 42 at. % aluminum is required. The β-phase appears at aluminum concentrations lower than 42 at. %, which significantly improves the heat treatment [20]. However, this phase has lower mechanical properties, so most applications requiring high heat strength must contain about 46 at. % aluminum [18]. If the TiAl46 alloy (at. %) is cooled sufficiently quickly from the α-phase (the rates are similar to thin castings), a lamellar structure is formed, and γ-phases precipitate on the crystallographic plane (0001). If the sample is heat-treated in the region of a two-phase structure, a duplex structure formed by γ-phase with lamellar grains is formed during cooling [18,21].

TiAl alloys have already begun to be used as turbochargers in commercial vehicles [4]. The development of TiAl alloy turbochargers began in Japan, when a relatively small number (about 1000) of TiAl turbochargers were installed in 1998 on Lancer cars [18]. In 1999, turbochargers were shown to Mitsubishi Motors Inc. for the model Lancer 6 [19] and great success in the following years led to the addition of more than 20,000 cars in 2003 with a TiAl alloy turbocharger [18]. Today, some companies now manufacture turbochargers based on TiAl, and it seems possible to successfully use a TiAl alloy in this relatively simple application, where very low toughness is acceptable to designers [18].

## 3. Properties of TiAl Alloys and Effect of Silicon on Them

As already mentioned, TiAl alloys excel in low density. Their other properties include good resistance to oxidation at temperatures of 600 to 800 °C [5,11], high specific strength at higher temperatures [22,23], and a favorable ratio between density and mechanical properties [12]. However, TiAl alloys have low ductility at room temperature (1–2%) [10,19,24] and fracture toughness [5,25]. Other properties of TiAl alloys are low thermal expansion and thermal conductivity comparable to nickel superalloys and refractory steels. The electrical conductivity is lower than that of commonly used alloys [26].

TiAl (50:50) alloy cannot be used above 827 °C, although it contains 50% aluminum, because the aluminum content is not enough to form a layer of alumina with protective effect. Approximately 75% aluminum is required to form the oxide layer [27]. Improvement of oxidation resistance can be achieved, for example, by surface modification or by adding silicon to TiAl powder in mechanical alloying [28]. By modifying the surface, silicon diffuses into the TiAl alloy, demonstrating the high affinity of silicon and titanium. The resistance to oxidation of TiAl alloys was also increased by the method of liquid phase siliconization with Al–Si alloy [28]. Silicon surface treatments are described, for example, in articles [29,30,31,32,33,34,35,36,37,38,39]. Siliconizing is a treatment to improve the chemical and mechanical properties of intermetallics based on TiAl system. Siliconizing methods can be used to obtain silicide layers on the surface of titanium-based alloys. These methods [30,32,34,35,36] combine in a siliconization process in the vapor phase and subsequent heat treatment [37], siliconization in the liquid phase combined with aluminizing in an Al–Si melt [19,38,40], heat treatment in quartz glass ampoules under low partial pressure of oxygen [39], or sol–gel coating in combination with heat treatment [30]. The silicide layers are very hard and prevent oxidation up to 950 °C.

The addition of silicon into the TiAl alloys improves oxidation and corrosion resistance at elevated temperatures, as well as creep resistance [41,42]. Silicon dissolves very little in TiAl alloys and therefore forms hard and brittle titanium silicides in the structure [17], mainly Ti_5_Si_3_ silicides, which have a strengthening effect [22,43]. A major problem of TiAl–Si alloys is the brittleness of titanium silicides, so they need to be as fine and fibrous as possible, which would increase the fracture toughness of the material [14]. Silicon is a light element, and the addition of more silicon to TiAl alloys improves the creep resistance and oxidation resistance due to the refinement of titanium dioxide particles formed on the surface of TiAl alloys. Silicon promotes the diffusion of aluminum in the oxidation layer and binds titanium to a stable silicide, then reduces the activity of Ti^IV^ and prevents the diffusion of Ti^IV^ outwards. The formation of titanium dioxide is thus limited [6]. The addition of silicon to TiAl alloys improves the oxidation resistance due to the formation of titanium nitride, which is formed in the form of a layer or isolated particles between the oxide layer and the base material and thus protects the base material from further oxidation [7].

High oxygen solubility in α-Ti (about 14.5 wt. %) leads to the formation of a brittle and hard subsurface layer enriched with oxygen, which cracks and causes deterioration of fatigue resistance and ductility [44,45,46,47]. Alloying by silicon improves oxidation resistance, oxide layer adhesion, and creep behavior at elevated temperatures [48,49]. High concentrations of silicon are more advantageous for improving resistance to oxidation than concentrations in the solubility range of silicon in α-Ti [50]. Kitashima et al. [51] showed similar oxidation behavior of an near-alpha alloy Ti-5Al-2Sn-2Zr-2Mo with 0.2 and 0.9 wt. % silicon [51]. However, commercial titanium alloys do not contain silicon in concentrations exceeding its solubility range in α-Ti (0.3 wt. %), which is mostly due to the negative effect of silicon on their ductility, especially when the silicon contents exceed 2 wt. % [52]. In addition, some researchers speculate that precipitation of silicides in aluminum-containing alloys may limit the formation of the heat-resistant phase Ti_3_Al, thereby reducing high temperature strength. Mechanisms that increase oxidation resistance include reducing the depth of oxygen penetration into the metal substrate and dissolving the silicon in the oxide surface layer. Silicon dissolved in the oxide layer reduces the rate of oxygen diffusion through the layer and modulates the stress relaxation that leads to a more compact and less porous oxide layer [49,50]. In addition, the formation of silica (SiO_2_) in the oxide surface layer inhibits the recrystallization of rutile (TiO_2_) crystals. In the work of Novák et al. [53], the addition of silicon and aluminum has been found to reduce the oxidation rate of titanium to approximately 850 °C. Silica is formed in the oxide layer on titanium. In silica, oxygen diffuses more slowly than in rutile (titanium dioxide). In addition, the almost continuous sub-layer of silicides is formed beneath the oxide layer as a result of the depletion by aluminum, which diffuses to the oxidized surface [53]. This layer acts as the secondary protection and suppresses the diffusion of both oxygen and nitrogen towards the core of the material. As a result, titanium nitride TiN is formed below the oxide layer. It has been proved that TiN is due to the presence of silicon in the material, because TiN formation was not observed in the binary alloy TiAl [7].

Several authors described the high temperature oxidation of intermetallic alloys based on TiAl system [54,55,56]. Titanium and aluminum diffuse into the surface in the early stages of oxidation and form a mixture of rutile and corundum. The formation of the oxide layer is controlled by diffusion, so the TiAl alloys exhibit parabolic behavior. Two factors affect the oxidation kinetics: (a) vacancies diffusion through the oxide layer and (b) formation of cracks, defects, and pores in the oxide layer [56]. The rate of TiO_2_ formation is higher than the rate of Al_2_O_3_ formation according to the kinetics of oxidation; therefore, titanium dioxide (rutile) is preferentially formed on the surface [54]. The formation of rutile on the surface leads to the formation of an inner layer rich in alumina (corundum). Concurrently, titanium diffusion to the surface could form the pores at the interface between the rutile-rich outer layer and the corundum-rich inner layers, thus making oxide delamination easier due to thermal stress due to the repeated heating and cooling. Under the alumina layer, the titanium-rich material again forms a layer of rutile. Therefore, the oxide layer is formed by a mixture of titanium dioxide and alumina [54,55]. In the article [57], the oxide layer on the surface is formed by rutile with corundum islands, under which there is a mixture of rutile and corundum. Below this layer is a titanium-rich region [57]. According to another article [58], the main component of the oxide layer should be alumina, as it has a lower Gibbs energy than rutile or silica (ΔG_f_ (800 °C, corundum) = −1073 kJ·mol^−1^, ΔG_f_ (800 °C, rutile) = −745 kJ·mol^−1^, ΔG_f_ (800 °C, silica) = −713 kJ·mol^−1^) [59]. However, the Gibbs energy per mole of reacting metal determines whether the metal will oxidize. Hence the Gibbs energy for corundum is −536 kJ·mol^−1^ and the Gibbs energy for rutile is −745 kJ·mol^−1^. So the oxide layers are formed mainly by rutile due to the high amount of titanium in the alloy and the more negative recalculated Gibbs energy for rutile. γ-Al_2_O_3_ transforms into δ-Al_2_O_3_ and α-Al_2_O_3_ is formed at high temperatures [58].

The Pilling–Bedworth ratio (PBR) expresses the ratio of the molar volume of the oxide layer and the atomic volume of the metal, equation:

(1) $$PBR=\frac{V_{ox}}{V_{k}}=\frac{\left(M_{ox}\cdot \rho_{k}\right)}{\left(n\cdot M_{k}\cdot \rho_{ox}\right)}$$

where *V_ox_* is the molar volume of the oxide layer, *V_k_* is the atomic volume of the metal, *M_ox_* is the molar mass of the oxide, *ρ_k_* is the density of the metal, *n* is the mass amount, *M_k_* is the atomic weight of the metal, and *ρ_ox_* is the density of the oxide.

If this ratio is less than 1, then the oxide layer is not compact and has no protective effects, because the formed oxide does not cover the surface compactly. At a ratio greater than 1, the layer may have protective properties. If the PBR reaches values higher than 2 or 3, the oxide layer is disturbed by stress or various defects, the layer cracks and delaminates, and its protective effect is reduced. For titanium and aluminum, the PBR is between 1 and 2 (1.73 for TiO_2_/Ti and 1.28 for Al_2_O_3_/Al [56]), which means that the sample is passivated and the protective oxide layer could protect the surface against further oxidation. However, in reality the rutile forms as porous and non-adherent at high temperatures and hence the protective properties of rutile itself are very poor [50]. In the case of silicon, the PBR is 2.15 for SiO_2_/Si [56], so that the oxide layer is delaminated, which does not protect the material from further oxidation.

The chemical and mechanical properties of TiAl alloys at high temperature are still insufficient. In this regard, research aims to improve these properties by: (a) modifying the alloy with alloying elements; (b) surface modification–formation of coatings on the surface of alloys [28,29,60,61,62]. Protective coatings are more effective than the alloying process, in which excessive amounts of alloying elements can adversely affect the final properties of the alloy. Often, elements that increase oxidation resistance reduce mechanical properties, so many authors resort to oxidation protection using TiAl_3_-rich barrier coatings [63].

Alloying and surface modifications increase the resistance to oxidation of titanium and titanium aluminides [6,64].

Shida et al. [27,65] classified the elements according to the effect on the resistance to oxidation of titanium aluminides. Among the harmful elements, he included, for example, vanadium, chromium, or manganese (which, however, improve ductility at room temperature); he included neutral zirconium, hafnium, tantalum, cobalt, nickel, and tin; and as suitable alloying elements he included niobium, molybdenum, tungsten, silicon, carbon, and boron [27,65]. Tungsten and molybdenum enrich the metal phase at the metal–layer interface, forming β-titanium rich in molybdenum or tungsten, in which aluminum diffusion can be rapid and oxygen solubility slow, leading to faster aluminum delivery and alumina formation on the surface [27]. According to Pilone et al. [64], on the contrary, chromium, niobium, silicon, and tungsten are suitable alloying elements for increasing the oxidation resistance [64].

The addition of cobalt as well as chromium increases the high-temperature resistance of TiAl-based alloys [22,25,28,43,66]. TiAl–Cr alloys are a promising material for protective coatings for γ–TiAl and α_2_–Ti_3_Al alloys due to the microstructure formed by the Laves phase of Ti(Cr,Al)_2_ [67,68]. The small addition of yttrium to TiAl-Cr based coatings increases resistance to oxidation up to 1000 °C [68]. Another research work has shown improved oxidation behavior of coating based on TiAl–Cr with the addition of 4 wt. % zirconium [69]. Brady et al. [70] proved that the addition of 8 to 10 at. % of chromium in TiAl alloys reduces the critical aluminum content required to form an alumina layer from 60 to 70 at. % to about half, thus improving oxidation resistance [70]. Chromium oxide acts as a diffusion barrier against ion transfer, which improves the resistance of TiAl intermetallic alloys at high temperatures [6,71,72,73]. The addition of niobium improves the high-temperature behavior of TiAl alloys [22,25,28,43] and increases the yield and tensile strength of TiAl alloys [7]. Niobium also reduces the oxygen solubility in the alloy, thereby limiting the internal oxidation of the material. Niobium promotes the formation of titanium nitride at the oxide layer–base material interface, which prevents the diffusion of oxygen and titanium ion [74]. Titanium nitride acts as a diffusion barrier that can prevent the diffusion of oxygen into the material. A layer of TiN is formed on the surface of the alloys in air at high temperature. Niobium has an effect on stabilizing titanium nitride, which can reduce the conversion of titanium nitride to titanium dioxide and reduce the oxidation rate of alloys [6]. TiAl–Nb alloys show excellent resistance to oxidation at 900 °C in air compared to TiAl alloy [74,75]. Yoshihara [76] confirmed that the most effective niobium content in TiAl alloy is 10–15 at. %. Excessive niobium content led to the formation of TiNb_2_O_7_ and AlNbO_4_ phases in the oxide layer, which in turn reduced the resistance to oxidation of the TiAl alloy at elevated temperatures. In addition, the high niobium content increases the density of titanium and titanium aluminides [76].

Even TiAl–Si alloys can be alloyed to improve mechanical properties, especially strength and ductility, or oxidation resistance. The most suitable alloying elements appear to be transition metals—cobalt, chromium, iron, molybdenum, niobium, and nickel. Jiang et al. [77] studied the effect of niobium (5–10 at. %) together with silicon (1–5 at. %) on the resistance to oxidation. The combination of niobium and silicon significantly improved the resistance to oxidation of alloys. In alloys with added silicon, a compact layer of Al_2_O_3_ is formed on the inside of the oxide layer. Increasing the niobium content in TiAl-based alloys prevents the growth of the Ti_5_Si_3_ phase, and also prevents the formation of TiO_2_ [77]. Vojtěch et al. [17] prepared an alloy TiAl39Si5Nb2 and found that niobium increases the strength and oxidation resistance of alloys and if the amount of niobium does not exceed 5%, it dissolves in TiAl and silicides [17].

In the work of Popela et al. [78] samples of Ti-46.6Al, Ti-45.2Al-7.2Nb, and Ti-44.8Al-6.6Ta were examined, which were siliconized in a powder of silicon and subsequently heated at 900, 1000, and 1100 °C for 2 to 12 h [78]. The kinetics of silicide formation were observed. The layer grew linearly with time at 900 °C; at 1000 and 1100 °C, the growth was suppressed after the first 2 h of siliconization [78]. The silicide layers formed at 900 and 1000 °C are composed of TiSi and TiSi_2_ sublayers, TiSi crystals with an elongated shape, and at 1100 °C the structure consists of a Ti_5_Si_3_ layer with an outer Al_2_O_3_ layer. Silicide layers have high hardness and improve oxidation resistance; in the case of ternary alloys the effect is even higher [78].

Nazmy et al. [79] developed an alloy Ti–47Al–2.1W–0.5Si (at. %), which has better resistance to oxidation and creep and its possible use was anticipated for marine turbines for high-speed ferries (maximum inlet temperature is 610 °C) [79].

## 4. TiAl–Si Alloys as a “CRM-Free” Material

TiAl–Si alloys have been developed as a replacement for materials containing critical metals (especially cobalt, tungsten, chromium, or niobium) [80,81]. In 2010, the European Union (EU) published a study on critical raw materials. A list of 14 critical raw materials (CRM) was created based on the economic importance and the risk of supply disruptions to the EU; in 2014, it already contained 20 critical raw materials, and in 2017 already 27 [80]. In 2017, silicon (metal) also appeared on the list; however, silicon obtained from recycling (e.g., of electronics or Al–Si castings from the combustion engines of passenger cars) is sufficient for TiAl–Si alloys [82]. In 2020, four more elements appeared on the list: bauxite, lithium, strontium, and titanium [82,83].

There are three solutions to the CRM situation: (a) improving raw material production processes (increasing sustainable extraction, reducing extraction costs, increasing material efficiency, etc.), (b) finding suitable substitutes to partially or completely replace critical materials, and (c) increasing recycling. The replacement of CRM should lead to innovative materials with comparable or better properties; materials should be easily and quickly integrated into production processes, with lower risks to the human health, environment, and lower prices [80]. Substitution of critical raw materials for use in extreme conditions is an important request, as extreme temperatures, wear, and corrosion often occur in many applications [80,84]. On the other hand, when some material is used as a substitute of a particular CRM, it increases its economic importance and it might become critical, which is probably the case of titanium. Oppositely, the substituted raw material could be potentially shifted out of the CRM list, so the CRM list will be always dynamic.

## 5. Preparation of TiAl–Si Alloys by Melting Metallurgy (MM)

The synthesis of the TiAl intermetallic compounds is one of the important directions in the development of new materials with heat resistance and high thermal stability [85]. Methods for producing TiAl alloys (and intermetallics in general) include conventional casting techniques, arc melting in an argon atmosphere, powder metallurgy, etc. [85].

The preparation of TiAl–Si alloy is very difficult using MM. This is due to the very high melting point of intermetallic compounds (e.g., titanium silicide Ti_5_Si_3_ melts at temperatures above 2100 °C [12,66]), high melt reactivity with crucibles [43], damage to crucibles (crucibles made of Y_2_O_3_ and ZrO_2_ are reported to be required [40], which is more expensive than graphite or corundum crucibles), and melt contamination. Other disadvantages of the preparation of TiAl–Si alloys by MM include very poor casting properties of intermetallics (very frequent occurrence of casting defects, such as pores or microcracks) [11]. In order to eliminate defects, the castings are post-processed by hot isostatic pressing (HIP). This technology is used by GE for GEnx engines for Boeing airliners [86].

The preparation of TiAl alloys with addition of silicon by MM is limited to hypoeutectic and eutectic alloys based on α_2_-Ti_3_Al, as hypereutectic alloys are extremely brittle due to the formation of coarse primary silicides during solidification [22]. It is necessary to design the material so that the composition of the alloys is around the eutectic point, so that the eutectic phases are fine. The eutectic composition is advantageous, because the meltability and fluidity are improved during casting [14].

Melting and casting of TiAl–Si alloys produce brittle and hard titanium silicides Ti_5_Si_3_, and these coarse and randomly oriented sharp-edged silicide particles are undesirable in terms of deterioration of some mechanical properties (especially fracture toughness) [7,24]. Previous work has sought to eliminate it and to form an in-situ composite of elongated Ti_5_Si_3_ silicide particles in a tough matrix of TiAl or Ti_3_Al titanium aluminides. However, there were cracks in the structure perpendicular to the solidification direction (due to the different thermal expansion coefficient of the Ti_5_Si_3_ titanium silicide in different crystal directions, therefore tensile stresses and crack initiation occurred). This results in a limited applicability of TiAl–Si alloys prepared by directional crystallization [7]. The stress in the material can generally be reduced by gradual slow heating and cooling, but for a polycrystalline material with high anisotropy of the coefficient of thermal expansion, it is not possible to reduce the residual stress [87].

**Figure 1:**
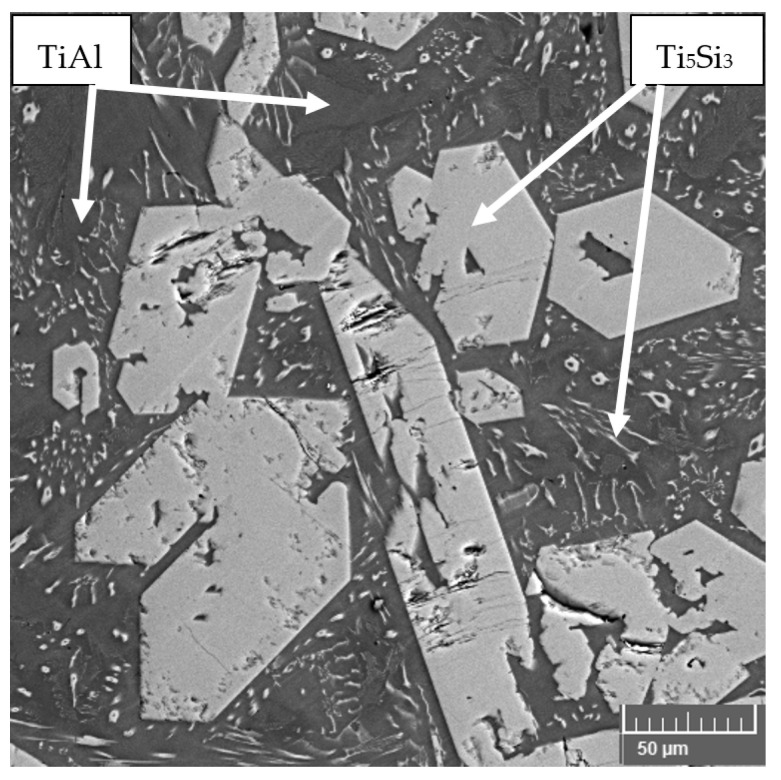
Microstructure of TiAl15Si15 prepared by vacuum induction melting.

TiAl–Si alloys prepared by MM have a very coarse microstructure with concentrated pores in the center of the material. The particles of titanium silicide, which are coarse, are oriented depending on the local direction of heat dissipation, which causes the elongated silicides, which was described in the article [17,87]. These silicides are cracked due to the high rate of cooling after melting process (Figure 1) [87]. The porosity in the center of sample is given by the production technology. The gases are trapped inside the material as a result of the elements evaporation (especially aluminum) during the exothermic reaction of intermetallic phases and the relatively fast cooling of the reacted sample. Gases cannot escape from the material due to the rapid solidification of the surface. Therefore, the porosity is concentrated in the center of the sample. The same applies to samples prepared by reactive sintering described, for example, in [14,88].

## 6. Preparation of TiAl–Si Alloys by Powder Metallurgy (PM)

The advantages of powder metallurgy include the acquisition of a finer and more homogeneous microstructure, which could cause an improvement in mechanical properties.

### 6.1. Reactive Sintering (RS)

Reactive sintering is a thermally activated process that produces compounds from elemental components, usually powders [43,89]. Reactive sintering usually results in very exothermic reactions, due to which there is no need to supply additional energy after initiation and the reaction spreads spontaneously, so it is sometimes referred to as self-propagating high-temperature synthesis [12,89,90]. The reaction, which changes the reactants to products, is exothermic, and the reaction rates may be high enough for adiabatic conditions to occur. All the heat of reaction raises the temperature of the material to an adiabatic temperature with nearly zero heat loss. The maximum experimental temperature reached during the reaction is called the combustion temperature [91].

Reactive sintering is used for the preparation of ceramics, intermetallic compounds, and their composites [11,89], but also for the preparation of borides, carbides, or nitrides [92]. The advantages of reactive sintering include energy savings [91], low temperatures and reaction time [91], higher purity of products [92], or evaporation of impurities with a low boiling point [90,91]. The disadvantage of reactive sintering is the porosity of the resulting material in many cases or the limited possibilities of process control (fast reaction, maximum temperature sustainable only for a short time, impossibility to change time) [91].

Self-Propagating High Temperature Synthesis (SHS) is divided according to the method of initiating to the “Thermal Explosion” (TE–SHS) and Plane Wave Propagation (PWP–SHS) reactions. These processes are characterized by high reaction temperatures (up to 4000 °C) and short reaction times (several seconds for TE–SHS and wave speed for PWP–SHS up to 250 mm/s) [93]. In the case of PWP–SHS, the compact reactant ignited at one end creates a reaction wave that consumes the reactants and produces products. If the reaction is initiated using an external heat source, the reaction is self-sustaining, using the heat of reaction to heat the adjacent reactant layer to the ignition temperature, and the wave propagates without the need for an external heat source until the entire material has reacted [91]. The second method of ignition is called thermal explosion (TE–SHS), in which the entire volume of the material is heated to the ignition temperature and all the reaction powder is spontaneously converted into a product. SHS converts powders to the desired compound [89]. The use of SHS in combination with the subsequent compaction and shaping of the product makes it possible to prepare various materials based on intermetallic compounds [85]. Both methods have been used in the past to produce nickel aluminides [91].

The RS of titanium aluminides is very well described. The initiation temperature for the formation of aluminides is given around the melting point of aluminum (660 °C). Aluminum is melted, filling the pores through capillary forces, thereby reducing the porosity of the product [7,89]. However, the product of reactive sintering of titanium and aluminum is still very porous. The reason for porosity may be Kirkendall porosity due to unbalanced diffusion (aluminum diffuses faster in titanium than titanium in aluminum), gas evolution due to evaporation of impurities on the powder particles surface (especially chlorides in titanium [94]), and the difference in density of the reactants and the product (TiAl has a density of 5.2% higher than the starting mixture of titanium and aluminum powders [95]) [91].

In Yang et al. [96], one end of the TiAl alloy sample was ignited at a temperature close to the melting point of aluminum. The reaction spread throughout the sample until it was completely transformed. The product was a porous material. This porous material can then be densified, for example, by hot pressing [96]. However, this consolidation method is very costly with low productivity, which is not suitable for commercial purposes [97]. Wenbin et al. [97] showed that during the RS of TiAl alloys, aluminum atoms move into the titanium lattice, leading to the formation of Kirkendall porosity, the porosity of the alloy before RS can be reduced by rolling, extrusion, or mechanical grinding [97].

The addition of silicon has a positive effect on the reaction between titanium and aluminum [11]. TiAl–Si powder mixtures react faster (within a few minutes) than TiAl alloys [12]. The mechanism and kinetics of reactive sintering of TiAl and TiAl–Si alloys were described in Novák et al. [12]. It has been shown that during reactive sintering, aluminum first melts, which causes an endothermic effect at 660 °C [12,14]. This was followed by a reaction between solid titanium and liquid aluminum to form the TiAl_3_ phase (this phase has a melting point of 1350 °C and a density of 3.3 g/cm^3^ [98]). Another significant exothermic effect signaled the formation of other TiAl phases. For the TiAl15Si15 alloy prepared from pure titanium, aluminum, and silicon powders (heating 10–30 K/min), only slight thermal effects were observed. In contrast, in the TiAl15Si15 alloy prepared from the Al–Si master alloy (heating by the rate of 10 K/min), an endothermic effect was observed at 580 °C, which was attributed to the formation of the Al–Si melt by eutectic transformation. The following two endothermic effects were the formation of titanium silicides and aluminides [12].

**Figure 2:**
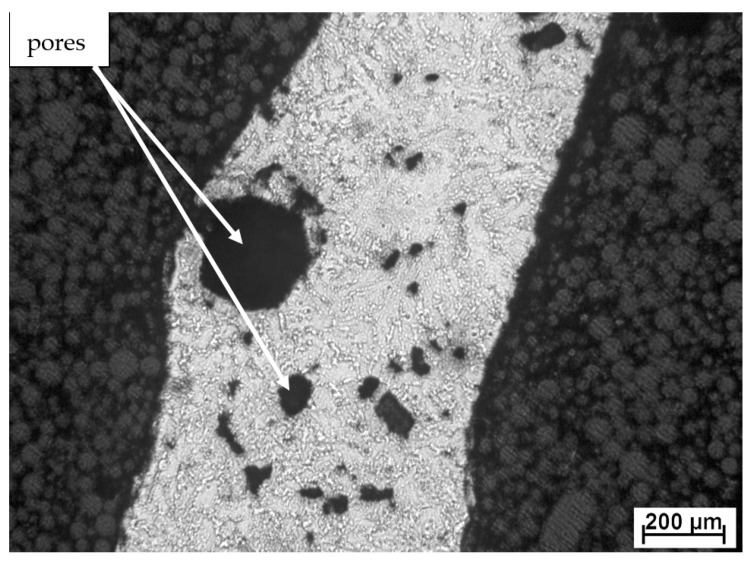
Microstructure of TiAl10Si30 after reactive sintering.

In the article [99], they studied the isothermal oxidation of TiAl–Si alloys prepared by RS. A strongly exothermic reaction of RS produces intermetallic phases below the melting point of the reactants [89]. Materials with porous structure are formed (Figure 2) [91,100,101,102]. Oxygen can go through the pores in the sample and cause internal oxidation. The reactive surface of samples with porous structure is much larger than their calculated surface [99]. Therefore, TiAl–Si alloys prepared by RS have lower resistance to high temperature oxidation than TiAl–Si alloys prepared by RS and subsequent spark plasma sintering (SPS). The differences between the porosities of TiAl–Si materials prepared by RS and RS with SPS are shown in our article [100].

In another article, the authors stated that the resistance to oxidation of TiAl–Si alloys depends on the silicon content in the material, the weight gain after oxidation process decreasing with increscent amount of silicon. TiAl–Si alloys, although very porous due to RS, have better resistance to oxidation than the reference binary alloy TiAl45 prepared by melting metallurgy [99,103].

### 6.2. Mechanical Alloying (MA)

Mechanical alloying is an energy-intensive process in which a mixture of powders of different metals or alloys is ground together in a grinding vessel to form a homogeneous alloy [104]. It is an effective process of achieving a very fine grain [105]. By mechanical alloying it is possible to synthesize a number of equilibrium and nonequilibrium phases of the alloy and to obtain powders with grain size in the order of nanometers and homogeneous dispersion of individual particles [106], supersaturated solid solutions, metastable crystalline and quasicrystalline phases, amorphous powders [105], nanocrystalline structures [15,104,106,107,108], or to form an alloy of elements that are immiscible at equilibrium [23]. Recently, emphasis has also been placed on the preparation of intermetallic alloys based on titanium and aluminum [109,110,111], ternary systems based on TiAl [22] and TiAl/Ti_5_Si_3_ composites [108].

The starting materials (powders) for MA are usually 1–200 μm in size and must be smaller than the grinding medium [104]. MA begins by mixing the powders in a selected ratio and placing them in a mill together with the grinding medium (usually steel balls); this mixture is then ground for the required time until the composition of each powder particle is the same as the proportion of elements in the powder base mixture. The mechanically alloyed powder is then heat treated to obtain the suitable microstructure and mechanical properties [104].

At the beginning of MA, metallic powder particles are relatively soft and tend to flatten and cold weld to form large particles (some are up to three times larger than the original particles [104]). Thus, the particle size of the powder increases in the initial phase and subsequently refracts. The reason for the reduction in particle size is strain hardening, which causes an increase in strength and a decrease in plasticity, which significantly increases the brittleness [112,113]. As a result of the intense high-energy interaction between the grinding balls and the powder, the ductile phase undergoes a continuous cycle of plastic deformation, fracture, and welding [105]. At this stage, fracturing predominates over the cold weld. This creates fresh surfaces, which helps further cold welding of powder particles. These processes are repeated several thousand times during MA in a high-energy ball mill [23,42,104,105,108]. The grinding time must be chosen so as to reach a steady state between cold welding and fracturing of the powder particle [104]. After grinding, the steady-state is reached when the welding speed is equalized, which tends to increase the average particle size and the crushing speed, which in turn leads to a decrease in particle size. Smaller particles are able to withstand deformation without breaking and are welded into larger particles. At this stage, each particle contains essentially all the starting components in the ratio in which they were mixed [104]. Fragmentation of powder particles leading to the formation of fresh surfaces, reduced distance between particles, increased defect concentration, and a slight rise in temperature contribute to MA [23]. During the mechanical alloying, large deformation is brought into the particles, which is exhibited by the presence of crystallographic disturbances, such as dislocations, vacancies, or a large amount of grain boundaries. The presence of this defective structure increases the solubility of the elements in the matrix [104].

From the previous results of the investigation of the MA process at the University of Chemistry and Technology in Prague (UCT), it can be concluded that the temperature reached at the point of contact between the powder, the sphere and the wall exceeds 650 °C. The temperature of 650 °C is required for the formation of some intermetallic compounds, such as the binary phases Fe-Al of Ni-Ti prepared in [114,115]. The required temperature for the formation of these phases was determined by the DTA method on pure metal powder mixtures [116]. Therefore, the energy including friction and the use of a high ball to powder ratio can be expected to be more than twice as high (approximately 400–500 W). Current high-speed mills reach an estimated power supply of about 400 W [117].

In the work of Novák et al. [115] the conditions for MA of high-temperature intermetallics were summarized. The high ratio of balls (steel balls of 20 mm in diameter) to powder (50:1, 70:1) transfers high kinetic energy to the powder particles, even at speed of rotation (400–600 min^−1^) [115]. The use of any lubricant reduces the forces of friction and the temperature of the powder. The reactions that lead to the formation of intermetallic phases from elemental metals are thermally activated. This means that it is advisable to avoid the use of lubricants in this MA process, as has been shown in reference [114].

TiAl–Ti_5_Si_3_ composites were prepared by mechanical alloying. In [118], it has been shown that silicon is an suitable candidate for the formation of the reinforcing component Ti_5_Si_3_ in a TiAl-based composite. The Ti_5_Si_3_ phase has high strength and good chemical and mechanical compatibility with TiAl matrices due to the fact that its thermal expansion coefficient is close to TiAl [106]. Based on these results, a new preparation method for the synthesis of Ti_5_Si_3_/TiAl composites has been described [119]. The novelty of this technology lies in the fact that the Ti_5_Si_3_ reinforcement will be formed from precursors in the form of metastable phases (using a TiAl–Si powder mixture prepared by MA) and then incorporated into a TiAl matrix [106].

In the work of Suryanarayana et al. [23], TiAl/Ti_5_Si_3_ nanocomposites were prepared using MA. It has been shown that the ultimate compressive strength (UCS) of binary γ–TiAl alloys with nanometer-sized grains is about 2600 MPa and decreases very rapidly to low values at temperatures above 500 °C. However, the strength decreases faster for ultrafine-grained than coarse-grained materials. This means that the smaller the grain size of the sample, the higher the strength and the bigger the rate of decrease in compressive strength at elevated temperature [23]. The ξ-Ti_5_Si_3_ and γ-TiAl phase composites with volume fractions of the ξ-Ti_5_Si_3_ phase ranging from 0 to 60% were made by mechanical alloying from pre-alloyed atomized γ-TiAl and TiSi powders. Compact composites were subsequently produced by hot isostatic pressing [23].

**Figure 3:**
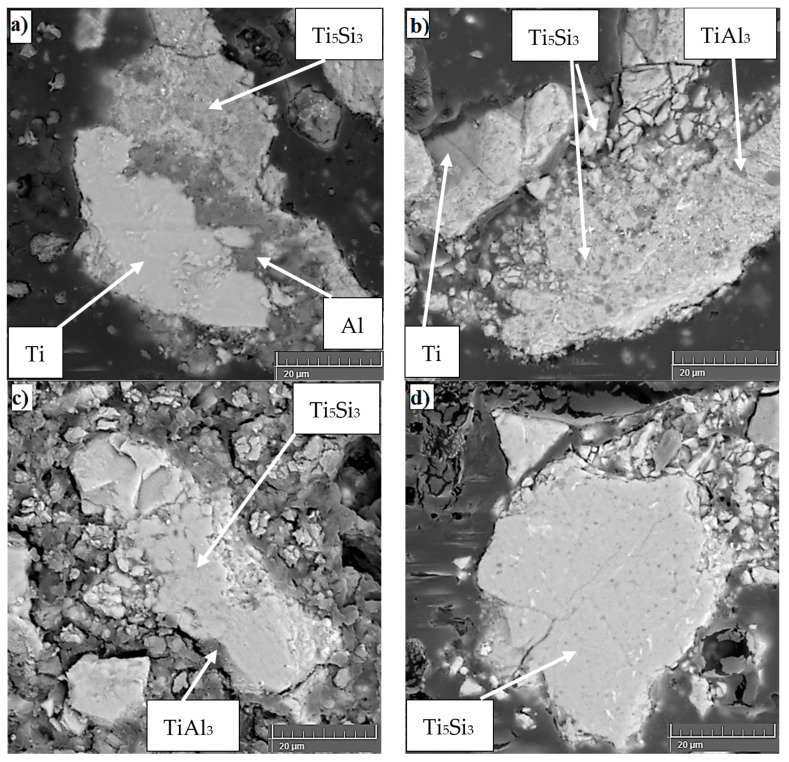
Microstructure of TiAl15Si15 alloy powders after MA: (**a**) 0.5 h, (**b**) 1 h, (**c**) 2 h, (**d**) 4 h.

In the work of Knaislová et al. [120], mechanical alloying of TiAl15Si15 alloys (wt. %) was tested (Figure 3) [120]. With increasing time MA of the alloy TiAl15Si15, it is possible to observe a preferred disappearance of pure elements of aluminum and silicon, while titanium is present in the powder even after 2 h MA. After 30 min, the first intermetallic phase of Ti_5_Si_3_ appears. This is due to the high mutual affinity of titanium and silicon, so titanium reacts preferentially with silicon. After 1 h of MA, all pure aluminum disappears and the first aluminide (TiAl_3_) is formed. The number of binary phases increases with increasing time, and after 4 h of mechanical alloying, the powder consists exclusively of intermetallic phases. MA causes the aluminum in titanium aluminides to be strongly substituted by silicon and the silicon in titanium silicides to be substituted by aluminum. The authors described that Ti(Al, Si) supersaturated solid solutions are formed after at least 10 to 20 h of grinding [106,108], but as shown in this work, intermetallic phases are formed during a much shorter mechanical alloying time (after 4 h) due to an optimization of the process conditions [120].

### 6.3. Spark Plasma Sintering (SPS)

Spark Plasma Sintering (SPS) is a modern method of powder compaction using compression while passing an electric current [7,121,122,123]. It is a technique using unidirectional pressure and direct pulsed electric current under low atmospheric pressure [124]. The SPS method is used to produce amorphous materials, intermetallic compounds, nanostructured materials, highly refractory metals and ceramics, or composites with a metal and ceramic matrix, which are difficult to prepare by conventional methods [121]. In the SPS process, heating rates of up to 1000 °C per minute can be achieved [121,124]. The heating rate depends on the geometry of the mold, the sample, and its electrical and thermal properties [124]. The sintering time usually reaches several minutes (usually a maximum of 10 min) depending on the sample, its dimensions, and the capacity of the equipment at low sintering temperature (sintering temperature is about 200 to 500 °C lower than most conventional sintering techniques [121,124]. With SPS, it is possible to consolidate a large amount of powder materials to a very high density in a short time [125]. One of the advantages of spark plasma sintering is rapid heating of the material and high thermal efficiency of the process [7,107]. Under pressure and pulse current flow, the temperature rises rapidly to 1000 to 2500 °C and the ambient temperature, leading to the production of a high quality sintered compact within a few minutes [124]. The high sintering rate at relatively low temperatures limits grain growth for most materials and the sintering efficiency is significantly increased compared to commonly used hot pressing (HP) and isostatic hot pressing (HIP) [107,121,126]. Other advantages over conventional sintering are, in addition to high sintering rate, high reproducibility, safety, and reliability [124].

**Figure 4:**
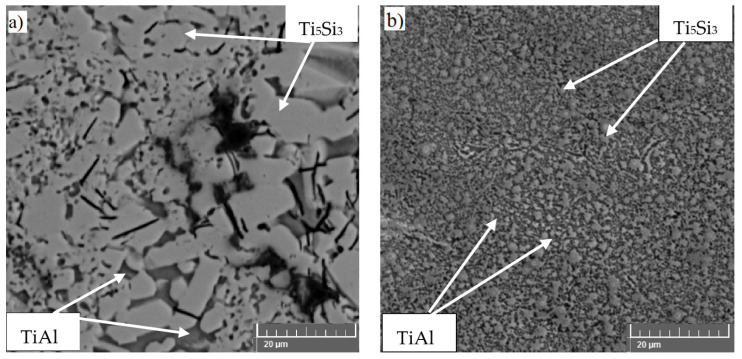
Microstructure of TiAl10Si20 prepared by: (**a**) reactive sintering + spark plasma sintering (RS + SPS), (**b**) mechanical alloying + spark plasma sintering (MA + SPS).

Spark plasma sintering method was tested on the two types of TiAl–Si powders, one was prepared by RS and the second one by mechanical alloying (Figure 4) [100,120,127]. TiAl–Si alloys prepared by RS and subsequent SPS compaction are characterized by a non-porous structure without visible unreacted starting components. The porosity of alloys reached about 2.5 vol. %. However, the prepared samples had very low fracture toughness, and there were cracks in the alloy. Cracks probably occurred during sample preparation, during cooling from the compaction temperature in the SPS process, because the cooling rate after compaction was higher than 200 °C/min. TiAl–Si alloys prepared by a combination of RS and SPS have indeed a significantly finer-grained and more homogeneous structure formed mainly by sharp-edged and unconnected particles of titanium silicides in an aluminide matrix than the same alloys prepared by MM. Despite a slow cooling rate from the compaction temperature, the cracks were found in the structure, especially in the titanium silicides. The ratio of the coefficient of thermal expansion along the *a* and *c* directions in the crystal structure of the titanium silicide Ti_5_Si_3_ is 2.7 [128]. Anisotropy of the coefficient of thermal expansion in polycrystalline titanium silicide causes internal stresses and cracking [129]. For this reason, MA followed by SPS with lower cooling rate was used. The aim of mechanical alloying was to significantly refine the microstructure, which was expected to positively affect the mechanical properties, including fracture toughness. By means of mechanical alloying, it is possible to further refine the microstructure, which would subsequently improve the mechanical properties as well as the fracture toughness. MA in combination with SPS led to a very fine microstructure of the TiAl–Si alloys. The authors have described that MA is a very efficient process for obtaining nanometer-sized grains [130,131]. The refinement of Ti_5_Si_3_ crystallites is probably due to strong deformation and subsequent recrystallization. The distribution of Ti_5_Si_3_ is much more homogeneous, because the brittle powder is crushed into very fine particles during the first step of the MA. The microstructure of TiAl–Si alloys prepared by MA followed by SPS is very homogeneous and fine-grained with contamination of iron from the milling vessel and milling balls. The porosity achieves values below 1 vol. %. The alloys have very high hardness, but fracture toughness is still low. The hardness, microhardness, and ultimate compressive strength of TiAl–Si alloys prepared by mechanical alloying followed by spark plasma sintering are higher than the same alloys prepared by melting metallurgy. The higher hardness and compressive strength correspond to a more homogeneous and less cracked microstructure. The force during MA plastically deforms the powder particles, which leads to strain hardening and fracture. Deformation reinforcements in combination with a very fine-grained structure increase the hardness of the material but reduce the plasticity [132]. High hardness at the expense of fracture toughness can be used in many applications, for example for tool materials. Abrasive wear resistance is very good for all alloys and comparable to tool steels. Wear resistance is closely related to the hardness and porosity of the material [88,130].

### 6.4. High Pressure Spark Plasma Sintering (HP SPS)

High pressure plasma sintering (HP SPS) was also tested. This innovative spark plasma sintering technology is used for difficult-to-sinter materials, such as polycrystalline diamond, refractory materials, cubic boron nitride, and ceramic composites [133,134,135,136,137], and has already been used for sintering ZrC-based composites [138]. A pulsed electric current heats the material from the outside as well as inside, which guarantees good powder compaction [136]. It is performed at very high pressure (up to 8 GPa) and very short time (tens of seconds) [133]. The short sintering time (maximum 3 min) eliminates the main problem of high-temperature processes, i.e., the tendency of grain growth in sintered material with increasing temperature and time [138,139,140]. Due to the high pressure, the process can also be applied to intermetallic compounds or minerals [141]. Brittle materials can acquire better mechanical properties after HP SPS due to the extended plastic deformation range [133].

**Figure 5:**
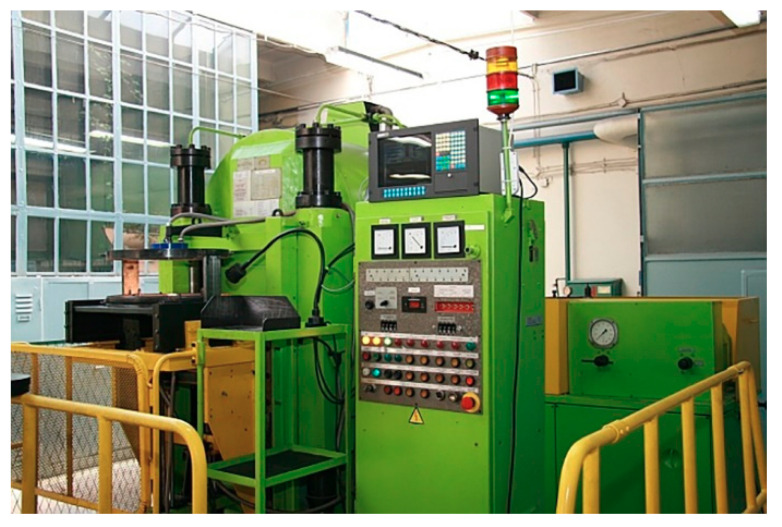
Device for high pressure spark plasma sintering [133].

**Figure 6:**
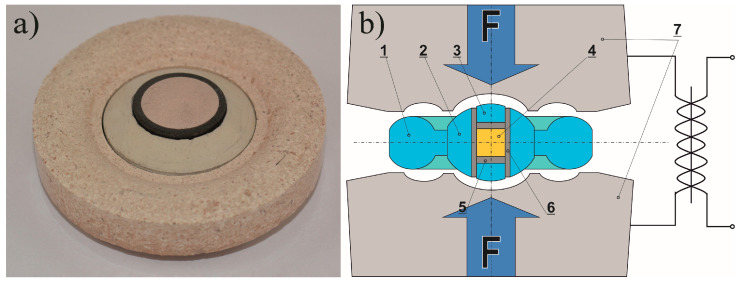
Figure 2 View of the high pressure gasket assembly (**a**) and cross-section diagram of the sintering process (**b**), where: 1—ceramic gasket (outer part); 2—ceramic gasket (inner part); 3—ceramic disc; 4—sample; 5—graphite disc; 6—graphite tube; 7—sintered carbide dies [133].

One of the equipment designs used for the HP SPS (Figure 5 and Figure 6) consists of a high-pressure hydraulic press equipped with a Bridgman anvil and a direct pulse current generator. The anvil has a toroidal shape, which helps to achieve quasi-isostatic compression of the material. Heating is performed with a pulse current of 1 kHz, which passes directly through the graphite heater into the conductive sintered material. The method has the main advantage of reducing the sintering temperature compared to conventional sintering methods. Another advantage of direct pulse current is derived from the use of very high heating and cooling rates and surface activation of powders by in-situ plasma cleaning, which can lead to the synthesis of new phases [121,133].

**Figure 7:**
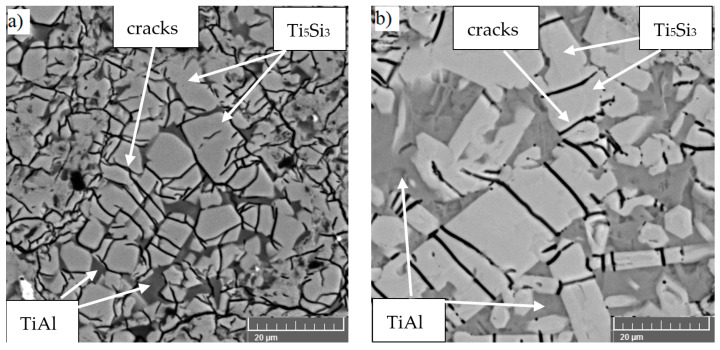
TiAl10Si20 alloy prepared by HP SPS: (**a**) 6 GPa, 907 °C, 150 s, (**b**) 6 GPa, 1324 °C, 30 s.

The goal of using this device was to take advantage of the possible huge compaction pressure (6 GPa versus 48 MPa in conventional sintering) to “force” the material to sinter better and interconnect the original powder particles, while reducing porosity to a minimum. Originally brittle materials could acquire better mechanical properties. It was found that phase composition of all tested TiAl–Si alloys is independent of compaction pressure. The microstructure (Figure 7) is heterogeneous with fine sharp-edged particles of Ti_5_Si_3_ in TiAl matrix. Cracks inside the titanium silicides are caused by the high pressure of compaction, very fast cooling rate from sintering temperature (approximately 100 °C/s), and probably also by the thermal expansion of sharp-edged silicides. The porosity of these alloys reached the values from 0.5 to 2 vol. %. The hardness increases with increasing silicon content (ratio of silicon to aluminum in the alloy), because silicon promotes the formation of silicides in the structure and the silicides have a higher hardness than the aluminides present. The hardness of all TiAl–Si alloys is comparable to tool steels. The ultimate tensile strength values and fracture toughness were low due to the cracks in the structure [133].

## 7. Effect of Silicon/Aluminum Ratio and Processing on the Phase Composition and Mechanical Properties

Table 1 shows a comparison of the phase composition of TiAl–Si alloys prepared by various technologies of melt and powder metallurgy. The phase composition of the TiAl10Si20 alloy prepared by melt and powder metallurgy is in accordance with the equilibrium phase diagram of TiAl–Si [100,120,142]. In addition, the silicides TiSi and Ti_5_Si_4_ were found in the TiAl10Si30 alloy due to the high amount of silicon in the alloy. The formation of TiSi and Ti_5_Si_4_ silicides corresponds to an equilibrium phase diagram [142]. TiSi silicide was not found in the TiAl10Si30 alloy prepared by MA + SPS, where it was probably converted into more stable silicides. The TiAl10Si30 alloy prepared by melting metallurgy, in contrast to the phase diagram, contained precipitated pure silicon but did not contain any aluminide. The TiAl15Si15 alloy has the same phase composition as corresponds to the phase diagram, although a small amount of titanium aluminide TiAl_2_ was still present in the alloy prepared by MA + SPS. According to the phase diagram, the TiAl20Si20 alloy is only two-phase, consisting of the most stable titanium silicide Ti_5_Si_3_ and titanium aluminide TiAl_3_, which is due to the higher ratio of aluminum and titanium not bound in silicides. However, after all types of preparation, the titanium silicide Ti_5_Si_4_ was also found in the TiAl20Si20 alloy.

The mechanical properties of the TiAl–Si alloys (Table 2) depend more on the processing technology than on the chemical composition. The alloys processed by arc melting reach the hardness in the order of 400–550 HV5, while all applied powder metallurgy processes increase this parameter to 800–1050 HV 5. The reason is probably in the refinement of the microstructure. In the case of the alloys processed by mechanical alloying, the deformation strengthening from the milling process also probably plays a role. Similar trends are also visible in the case of the ultimate compressive strength, where the alloys processed by mechanical alloying and spark plasma sintering reach higher strength values. Independently of the processing technology, the TiAl15Si15 alloy reaches the highest ultimate compressive strength due to a balanced ratio between hard silicide particles and relatively tough aluminide matrix. The presence of cracks (due to the anisotropy of the coefficient of thermal expansion of titanium silicides) has a great influence on the fracture toughness; therefore, the low fracture toughness of TiAl–Si alloys was not increased either by slow cooling from the compaction temperature or by refining the microstructure by mechanical alloying. The fracture toughness values were at most around 1 MPa·m^1/2^.

## 8. Conclusions

In this paper, the development of TiAl–Si alloys in the period 2003–2020 was summarized. The described alloys are in fact the in-situ composites, consisting of titanium aluminide (TiAl, Ti_3_Al, or TiAl_3_) matrix reinforced by hard titanium silicide (especially Ti_5_Si_3_) particles. The alloys are characterized by high hardness, wear resistance comparable with AISI D2 tool steel, and very good oxidation resistance at high temperatures (up to 1000 °C), but also low room-temperature ductility. Even though the alloys have been prepared by powder metallurgy (reactive sintering and mechanical alloying in combination with spark plasma sintering) and melting metallurgy (arc melting, vacuum induction melting), none of these methods was able to eliminate the room-temperature brittleness completely. Higher toughness was achieved in the case of the melting-metallurgy prepared alloys due to the coarse-grained structure. The cracks propagate in the silicide phase easily and stop in the aluminide phase. Future application can be found in the case of the parts’ loaded statically high temperature or coatings for high temperature and wear conditions, particularly valves of combustion engines, parts of jet engines, or protective coatings for power plants.

## Figures and Tables

**Table 1:** Comparison of the phase composition of TiAl–Si alloys.

Alloy	Phase Diagram [142]	Melting [143]	HP SPS [133]	RS + SPS [100]	MA + SPS [127,144]
TiAl10Si20	Ti_5_Si_3_, TiAl	Ti_5_Si_3_, TiAl	Ti_5_Si_3_, TiAl	Ti_5_Si_3_, TiAl	Ti_5_Si_3_, TiAl
TiAl10Si30	TiSi, Ti_5_Si_4_, TiAl_3_	TiSi, Ti_5_Si_3_, Ti_5_Si_4_, Si	TiSi, Ti_5_Si_3_, Ti_5_Si_4_, TiAl	TiSi, Ti_5_Si_3_, Ti_5_Si_4_, TiAl	Ti_5_Si_3_, TiAl, TiAl_2_, Ti_5_Si_4_
TiAl15Si15	Ti_5_Si_3_, TiAl	Ti_5_Si_3_, TiAl	Ti_5_Si_3_, TiAl	Ti_5_Si_3_, TiAl	Ti_5_Si_3_, TiAl, TiAl_2_
TiAl20Si20	Ti_5_Si_3_, TiAl_3_	Ti_5_Si_3_, TiAl_3_, Ti_5_Si_4_	Ti_5_Si_3_, TiAl_3_, Ti_5_Si_4_	Ti_5_Si_3_, TiAl_3_, Ti_5_Si_4_	Ti_5_Si_3_, TiAl_3_, Ti_5_Si_4_

**Table 2:** Comparison of the mechanical and tribological properties of TiAl15Si15 alloy.

Alloy	Hardness HV 5	Fracture Toughness [MPa·m^1/2^]	Ultimate Compressive Strength [MPa]
TiAl10Si20 melting [143]	416 ± 23	0.90 ± 0.52	647
TiAl10Si30 melting [143]	549 ± 49	1.03 ± 0.51	941
TiAl15Si15 melting [143]	421 ± 40	0.75 ± 0.51	1705
TiAl20Si20 melting [143]	463 ± 54	1.39 ± 0.32	643
TiAl10Si20 HP SPS [133]	865 ± 125	0.76 ± 0.20	530
TiAl10Si30 HP SPS [133]	1035 ± 130	0.61 ± 0.19	677
TiAl15Si15 HP SPS [133]	818 ± 70	1.16 ± 0.28	1314
TiAl20Si20 HP SPS [133]	818 ± 90	0.62 ± 0.25	931
TiAl10Si20 RS + SPS [100]	810 ± 61	1.23 ± 0.22	2103
TiAl10Si30 RS + SPS [100]	977 ± 58	0.89 ± 0.19	1255
TiAl15Si15 RS + SPS [100]	736 ± 32	1.40 ± 0.35	1963
TiAl20Si20 RS + SPS [100]	819 ± 69	1.03 ± 0.17	1564
TiAl10Si20 MA + SPS [120]	1015 ± 45	0.55 ± 0.05	2125
TiAl10Si30 MA + SPS [120]	1037 ± 51	0.50 ± 0.04	2262
TiAl15Si15 MA + SPS [120]	865 ± 42	0.31 ± 0.09	1908
TiAl20Si20 MA + SPS [120]	800 ± 98	0.40 ± 0.09	1918

## Data Availability

This is the review paper with minor amount of unpublished results of the authors. These data are stored by the authors, not available publically.
